# Exploring the Utility of Renal Resistive Index in Critical Care: Insights into ARDS and Cardiac Failure

**DOI:** 10.3390/biomedicines13020519

**Published:** 2025-02-19

**Authors:** Giuseppe Cuttone, Giulio Geraci, Luigi La Via, Massimiliano Sorbello, Federico Pappalardo, Caterina Carollo

**Affiliations:** 1 Faculty of Medicine and Surgery, Kore University, 94100 Enna, Italy; giuseppe.cuttone@unikore.it (G.C.); massimiliano.sorbello@unikore.it (M.S.); 2 Department of Anaesthesia and Intensive Care 1, University Hospital Policlinico “G. Rodolico-San Marco”, 95123 Catania, Italy; luigilavia7@gmail.com; 3 UOC Intensive Care, Hospital “Giovanni Paolo II”, 97100 Ragusa, Italy; 4 Department of Anesthesia and Intensive Care, University Hospital, Heart Centre “GB Morgagni”, 95125 Catania, Italy; federico.pappalardo@unikore.it; 5 Department of Health Promotion, Mother and Child Care, Internal and Specialistic Medicine (PROMISE), Unit of Nephrology and Dialysis, “P. Giaccone” University Hospital, 90146 Palermo, Italy; caterina.carollo@unipa.it

**Keywords:** renal resistive index, critical care, ARDS, heart failure, duplex Doppler sonography

## Abstract

The renal resistive index (RRI), a Doppler ultrasound-derived parameter measuring renal vascular resistance, has emerged as a promising non-invasive tool to evaluate renal hemodynamics in critically ill patients, particularly those with acute respiratory distress syndrome (ARDS) and heart failure (HF). This narrative review examines the current evidence for RRI measurement in these conditions, exploring its physiological bases, methodology, clinical applications, and limitations. In ARDS, RRI reflects the complex interactions between positive pressure ventilation, hypoxemia, and systemic inflammation, showing a role in predicting acute kidney injury and monitoring response to interventions. In HF, RRI is able to assess venous congestion and cardiorenal interactions and can also serve as a prognostic indicator. Many studies have shown RRI’s superiority or complementarity to traditional biomarkers in predicting renal dysfunction, although its interpretation requires consideration of multiple patient-related factors. Key challenges include operator dependency, lack of standardization, and complex interpretation in multi-organ dysfunction. Future research should focus on measurement standardization, development of automated techniques, investigation of novel applications like intraparenchymal renal resistive index variation, and validation of RRI-guided management strategies. Despite its limitations, RRI represents a valuable tool that offers bedside and real-time insights into renal hemodynamics and potential guidance for therapeutic interventions. Further research is needed to fully clarify its clinical potential and address current limitations, particularly in critical care settings involving multiple organ dysfunction.

## 1. Introduction

Renal resistive index (RRI), an ultrasound vascular Doppler-derived parameter, evaluates renal resistance and compliance; it was first described by Pourcelot in 1974 [1,2].

RRI was primarily explored as a diagnostic tool in situations in which intrarenal impedance is altered, particularly renovascular and chronic kidney disease; however, it has recently been reported in critical care, where it may be able to capture changes in renal perfusion and predict acute kidney injury [3,4].

Adequate organ perfusion is fundamental in critically ill patients. Intrarenal circulation is sensitive to fluctuations in systemic hemodynamics, and such changes frequently mark an early sign of systemic circulatory afterload [5]. Serum creatinine and urine output are indirect markers of the physiologic processes governing renal function; however, they are late and low-sensivity indicators of acute kidney injury. On the contrary, RRI provides real-time information about renal hemodynamics, which may result in earlier recognition of renal dysfunction in acute settings and earlier interventions [6].

The coordinated interplay of positive pressure ventilation, hypoxemia, and systemic inflammation exerts a direct influence on renal function in acute respiratory distress syndrome (ARDS) [7]. The renal resistance index may potentially serve as an indicator of these complex interactions, thereby offering a novel marker of renal hemodynamics. Similarly, in heart failure (HF), RRI may reflect the interrelationships between cardiac output, venous congestion, and renal perfusion [8].

The aim of this narrative review is to evaluate the existing evidence regarding the use of RRI in both ARDS and HF. We will explore the physiological mechanisms underlying its relevance, the techniques employed for its measurement, its clinical applications, and its prognostic value while also addressing its limitations and considering future research directions.

## 2. Measuring RRI: Methodology

**Figure 1:**
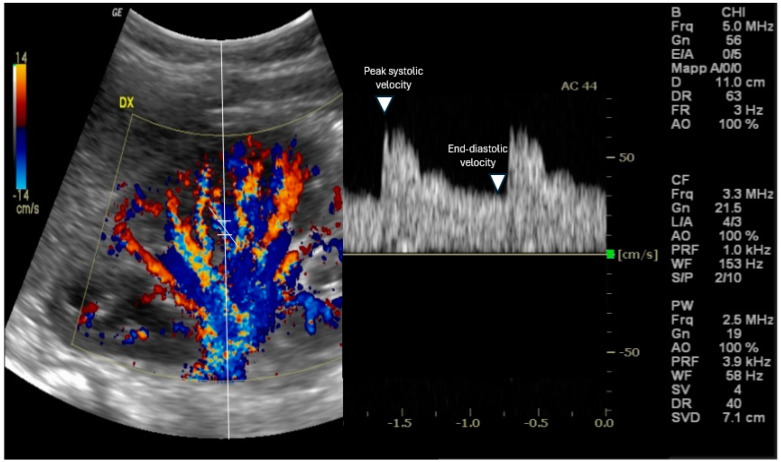
Evaluation of the renal resistive index using Doppler ultrasound. The transducer is placed in an interlobar artery, and the spectral Doppler examines the peak systolic and end-diastolic velocities.

Doppler ultrasonography is a non-invasive imaging technique that enables real-time visualization of blood flow in renal vessels [9]; RRI is assessed using Doppler ultrasonography. The procedure usually occurs with the patient typically either in the supine position or in a slight lateral decubitus position to provide optimal exposure of the kidneys [10]. The kidney is identified, and its anatomy is evaluated via B-mode ultrasound, then, color Doppler augmentation allows for the detection of the main renal artery and its branches inside the kidney [11] [Figure 1].

This usually includes three types of Doppler to obtain flow information, to visualize subtle and slow blood flow, and to show blood flow velocity over time as a waveform. Usually, a lower frequency, the curvilinear transducer, is preferred (typically 3.5 to 5 MHz) in adult patients since both renal arteries and kidneys are in a deep location. A pulsed wave Doppler sample volume is located in an interlobar or arcuate artery at the corticomedullary junction in most cases [12] in addition to the peak systolic velocity [PSV] and end-diastolic velocity [EDV] measurements from the arterial waveform. Therefore, RRI is calculated using the formula: RRI = (peak systolic velocity − end-diastolic velocity)/peak systolic velocity [2]. At least three measurements should be taken from separate interlobar arteries in each kidney, and the mean value is considered for the final RRI [6].

To ensure the reliability and reproducibility of RRI measurements, the following parameters should be considered:

- Skills: RRI measurement needs a trained operator [13] to achieve precise and repeatable results.
- Standardized ultrasound machine settings, including Doppler gain, pulse repetition frequency, and wall filter should be used to minimize variability [14].
- Measurement site: performing measurements in the same location [i.e., always at the corticomedullary junction] is better for comparison [15].
- Patient factors: breath-holding, heart-rate, and blood pressure can influence RRI and should be standardized or controlled accordingly [16].
- Time of assessment: for studies involving critically ill patients, RRI should be assessed after any intervention, as this will differ in terms of best practice [17].
- Inter- and intra-observer variability: these might depend on various factors, as discussed above [18].
- Reporting standards: this is a crucial factor for comparability between studies and implementation into clinical practice [19].

Incidentally, bedside RRI measurement is relatively fast and simple, so it can be performed in most critical care units. Nevertheless, the awareness of methodological issues is a prerequisite for the appropriate interpretation and use of RRI in such a context.

## 3. RRI in ARDS and HF: Pathophysiological Bases

Under physiological conditions, the kidneys are especially sensitive to variations in systemic hemodynamics [5], receiving about 20–25% of cardiac output. The critical illness, for example, influences renal perfusion, resulting in changes in output, systemic vascular resistance, and intravascular volume status [20].

In patients with ARDS [21], renal function and intrarenal hemodynamics are significantly influenced by the complex interaction between hypoxemia, inflammatory mediators, and positive pressure ventilation. Moreover, hypoxemia causes renal vasoconstriction as a compensatory mechanism to preserve oxygen delivery to other organs; however, prolonged hypoxemia creates cellular injury and oxidative stress, which are both detrimental to renal function [22]. Mechanical ventilation, one of the cornerstones of ARDS management, might considerably influence renal hemodynamics and, in turn, RRI: mechanical ventilation produces positive intrathoracic pressure that decreases venous return and may decrease cardiac output and renal perfusion [23]. Mechanical ventilation is able to both increase right atrial pressure, which in turn, may increase renal venous pressure, and decrease the trans-renal perfusion pressure gradient [24], stimulating neurohumoral mechanisms such as the renin-angiotensin-aldosterone system that influences renal vascular resistance and intrarenal circulation [25]. These effects are magnified at higher levels of positive end-expiratory pressure [PEEP] and in patients with baseline cardiac dysfunction [26]. With the initiation of mechanical ventilation, RRI has been shown to increase, and such an increase has been positively correlated to the degree of PEEP [27], suggesting that it could reflect the pathophysiologic response to changes in intrathoracic pressure, cardiac function, and renal perfusion.

Both diminished cardiac output and increased venous pressure can result in changes in renal perfusion in cardiac failure [28]. Low cardiac output results in decreased renal perfusion while declining renal perfusion increases renal vascular resistance as a compensatory mechanism [29] and increased central venous pressure [especially in right HF] leads to renal congestion. Taken together, these changes cause increased renal interstitial pressure, thus affecting renal blood flow and glomerular filtration [30,31].

Consistent with these findings, RRI has been shown to correlate with cardiac function biomarkers like left ventricular ejection fraction and right atrial pressure in patients with both acute and chronic heart diseases [32,33]. Whereas neurohormonal activation [sympathetic nervous system and renin-angiotensin-aldosterone system] may contribute to further increases in renal vascular resistance [34], changes in RRI correlate with improvement in cardiac function with treatment, indicating that RRI may be predictive of response to therapy [35].

## 4. RRI in ARDS

ARDS is an extreme form of acute lung injury characterized by systemic inflammation, impairing gas exchange and typically inducing the need for mechanical ventilation. The complexity of ARDS pathophysiology can deeply influence renal physiology. In this context, RRI deserves greater attention as a non-invasive tool to assess renal perfusion and AKI susceptibility.

Many studies have found an association between RRI and the severity of ARDS, especially in COVID-19 patients [36,37,38]. In addition, Darmon et al. found that RRI values were significantly higher in patients with moderate to severe ARDS compared with mild ARDS or no ARDS patients [27]. This association may be due to physiologic and pathophysiologic effects associated with severe lung injury, such as increased levels of inflammatory mediators and shifting hemodynamics that can affect renal perfusion.

In addition, RRI has also been associated with oxygenation variables in ARDS patients. Lahmer et al. [39] found a strong inverse relationship between RRI and the PaO_2_/FiO_2_ ratio, a well-established marker of the degree of oxygenation impairment in ARDS. This suggests that RRI might become an additional marker of disease severity, and this could potentially help in monitoring the progression or improvement of ARDS.

During the COVID-19 pandemic, RRI emerged as a valuable tool for monitoring kidney function in critically ill patients. Fogagnolo et al. [36] demonstrated that COVID-19 patients under mechanical ventilation showed elevated RRI values, which were associated with worse respiratory parameters and increased mortality. These findings aligned with Giustiniano and coworkers’ hypothesis that RRI could serve as an early marker of inflammation in lung–kidney crosstalk, particularly relevant in COVID-19-related ARDS [37]. Garcia Cruz et al. [38] further validated RRI’s prognostic value, showing that higher RRI values on admission were independently associated with both the development of acute kidney injury and increased mortality in COVID-19 patients, suggesting its potential role as an early warning tool in critical care settings.

Among the possible applications, RRI could become a predictor of AKI in ARDS. Oliveira et al. [17] showed that high RRI levels predicted a more than double risk of AKI in critically ill patients, including those with ARDS, and found that a cut-off of 0.69 had a sensitivity of 85% and specificity of 79% in predicting AKI within 72 h.

RRI has not only been valuable in predicting AKI but also other important outcomes in patients with ARDS [11].

An elevated RRI has been independently associated with the development of AKI in critically ill patients, including those with ARDS. The prognostic significance of RRI in managing ARDS has been highlighted by various studies, underscoring its potential utility in clinical decision-making. Furthermore, the ability of RRI to reflect renal hemodynamics in response to changes in ventilator settings and fluid management enhances its promise as a marker for guiding ARDS treatment [40]. A recent retrospective study demonstrated that alterations in positive end-expiratory pressure (PEEP) were mirrored by corresponding changes in RRI, with increases in PEEP resulting in an elevated RRI and decreases in PEEP leading to a reduction in RRI. This finding supports the use of RRI to titrate PEEP, potentially minimizing nephron exposure to suboptimal renal perfusion.

Fluid optimization remains a critical aspect of clinical practice in ARDS management [41], and RRI could be an invaluable tool for guiding clinicians in preventing fluid excess without compromising organ perfusion.

Schneider et al. [42] recently showed that changes in RRI in response to fluid challenges could predict fluid responsiveness in critically ill patients, suggesting that RRI may be used to guide fluid administration in ARDS patients. This approach could help maintain renal perfusion while avoiding the risks associated with fluid overload. Main studies that have evaluated the prognostic significance of RRI in ARDS are presented in Table 1.

## 5. RRI in Heart Failure

HF is a heterogeneous clinical syndrome defined by the heart’s inability to maintain sufficient cardiac output (CO) to satisfy the body’s demands. HF may affect renal function through numerous mechanisms. RRI has proven to be useful for the evaluation of renal hemodynamics in patients with HF, providing information on complex cardiorenal interactions and possible prognostic data.

RRI is a multidimensional value in association with heart function in patients with HF. It has been shown that RRI is significantly related to CO and left ventricular function [32].

In chronic HF patients, a high value of RRI was independently correlated with low left ventricular ejection fraction (LVEF), and the same results were found in patients with coronary heart disease [33]. This relationship probably reflects the effects of decreased cardiac output on renal perfusion, resulting in increased renal vascular resistance as a compensatory response. This concept has been further validated by Darabont et al. [43], who extensively reviewed the significance of RRI in both kidney and cardiovascular disease.

The latest ESC guidelines for acute and chronic heart failure have introduced two new drug classes into medical treatment: sacubitril/valsartan and sodium–glucose co-transporter 2 (SGLT2) inhibitors [44]. The beneficial effects of sacubitril/valsartan in patients with heart failure and reduced LVEF extend beyond cardiac protection—encompassing natriuretic and diuretic properties, enhanced ejection fraction, reverse remodeling, and improved diastolic function—to also include positive renal outcomes, contributing to the management of cardiorenal syndrome [45]. By inhibiting angiotensin II receptors and neprilysin, sacubitril/valsartan promotes natriuresis and diuresis in the kidneys, leading to afferent arteriole dilation, improved glomerular filtration rate (GFR), and enhanced renal arterial flow, ultimately resulting in a significant reduction in renal resistive index (RRI) from 0.67 to 0.649. Furthermore, neprilysin inhibition reduces renal fibrosis by preventing efferent arteriole dilation, glomerular hypertrophy, and mesangial tissue expansion, thereby offering additional nephroprotective benefits.

Notably, in recent years, there has been much interest in the association between the right ventricular (RV) function and RRI. Husain-Syed et al. [46] showed that RRI is independently and strongly associated with RV dysfunction indices like tricuspid annular plane systolic excursion (TAPSE) and RV fractional area change. We focus here on this relationship, which is especially pertinent as RV function is critical to renal perfusion, particularly in the setting of high central venous pressure (CVP) common in right HF.

In HF, one of the most exciting potential uses of RRI is as a marker of venous congestion. Elevated central venous pressure has become increasingly recognized as an important mechanism of renal injury contributing to cardiorenal syndrome, manifesting as venous congestion. Based on the transition between euvolemia and hypervolemia in HF patients, Nijst et al. [8] showed that RRI rises substantially, thus suggesting that RRI could be a sensitive indicator of escalating venous congestion. This association of RRI with venous congestion is especially relevant when classic volume markers (e.g., central venous pressure measurement or clinical examination) could be unreliable or invasive. Recently, Fotopoulou et al. [47] demonstrated that RRI on ICU admission correlates well with tissue hypoperfusion indices, underlining its value as a non-invasive monitoring tool.

In HF patients, a few studies have shown the prognostic significance of RRI. Ciccone et al. [35] showed that RRI was an independent predictor of HF progression in chronic HF patients and specifically that patients with abnormal RRI had a significantly higher risk of HF hospitalizations and mortality. Moreover, in another study, Ennezat et al. [48] proved that RRI could predict negative outcomes in patients with decompensated HF who were admitted to the hospital and even found that an RRI ≥ 0.70 could predict increased risk of all-cause mortality and rehospitalization for HF. We proposed that the prognostic value of RRI in HF can be explained by the capacity of RRI to combine information on cardiac function, renal circulation, and systemic hemodynamics [49,50]. Therefore, it offers additional insight into the global cardiovascular health of the patient, possibly providing more comprehensive risk stratification than conventional markers alone [51]. This concept has been recently explored by Tomii et al. [52] in both heart failure with reduced and preserved ejection fraction and by Barone et al. [53] in patients undergoing coronary angiography, confirming RRI’s role as an independent predictor of worsening renal function.

RRI could serve as a valuable tool in fluid management, particularly in the context of acute decompensated HF. By providing insights into renal perfusion and venous congestion, RRI could enable clinicians to not only optimize volume status but also minimize renal dysfunction, thereby enhancing patient outcomes. Additionally, RRI holds potential as a marker for monitoring the response to HF therapy. For instance, in the study by Tudoran M. and Tudoran C. [54], improvements in RRI were associated with clinical success in the treatment of acute HF, with a decrease in RRI values suggesting treatment efficacy. Finally, RRI also plays a prognostic role, aiding in clinical decision-making for high-risk patients by providing crucial information about the patient’s renal and hemodynamic status. Recent technological advances have also enabled novel applications, such as the ultrasonographic intraparenchymal RRI variation described by Samoni et al. [55] for assessing renal functional reserve in cardiac surgery patients and the use of RRI in patients with left ventricular assist devices, as reported by Barua et al. [56]. Main studies that have evaluated the prognostic significance of RRI in HF are presented in Table 2. 

## 6. Relationship of RRI with Other Organ Dysfunction Markers

As previously stated, in critical care settings, RRI has been recently validated as a promising marker of renal impairment. However, to assess its overall clinical value, RRI needs to be compared with other more validated organ dysfunction markers, both serum biomarkers and other ultrasonography detected parameters.

Serum creatinine and blood urea nitrogen (BUN) are traditional cornerstones in the evaluation of renal function. Nevertheless, they exhibit limitations such as the time-lag appearance of the markers in AKI and they can be influenced by conditions independent of renal function. By comparison, RRI assesses renal hemodynamics in real time, enabling earlier identification of renal dysfunction.

Several studies have compared RRI with novel biomarkers of kidney injury [57,58].

Patel et al. showed that although serum cystatin C can predict AKI in critically ill patients, neither the sensitivity nor the specificity was as good as RRI. An RRI ≥ 0.70 was found to have a sensitivity of 75% and specificity of 84% compared with cystatin C with a respective sensitivity of 63% and specificity of 80% to predict AKI [58].

Another promising biomarker for early AKI diagnosis is neutrophil gelatinase-associated lipocalin (NGAL). In a group of septic patients who were not receiving antibiotics, Schnell et al. [11] compared RRI with plasma NGAL in the prediction of AKI and found that both markers were predictive of AKI, although RRI had a higher area under the receiver operating characteristic curve (AUROC) than NGAL [0.87 vs. 0.80]. Nevertheless, these biomarkers and RRI have a different prognostic value. While biomarkers such as NGAL and cystatin C are related to cellular damage in the kidney, RRI provides information on renal hemodynamics. Hence, the combination of RRI and these biomarkers may yield a more accurate assessment of renal function than either biomarker or RRI alone.

Besides RRI, additional ultrasonographic parameters have been investigated for organ dysfunction assessment in critical care. For example, plenty of these parameters are included in the venous excess ultrasound (VExUS) score, which evaluates venous congestion through the examination of inferior vena cava (IVC) diameter and hepatic, portal, and intrarenal venous flow patterns [59,60,61]. Beaubien-Souligny et al. [62] have compared RRI vs. the standard VExUS score for prediction of AKI following cardiac surgery. Both parameters were predictive for AKI; however, the combination of RRI and VExUS score was associated with a better predictive accuracy than either parameter alone. Echocardiography, as a specialist skill, is another ultrasonographic parameter commonly encountered in critical care, particularly with respect to assessment of left ventricular function. Nguyen et al. [63] compared the relationship between RRI and echocardiographic parameters and AKI in patients suffering from septic shock; both RRI and LVEF predicted AKI; however, for RRI, the AUROC was higher [0.86 vs. 0.72]. Lung ultrasound is a key ultrasonographic tool in critical care to detect extravascular lung water as well as in the diagnosis of conditions such as ARDS. Direct comparisons of lung ultrasound findings with RRI are limited; however, both provide information regarding fluid status and organ dysfunction. Additional studies comparing these parameters should be performed.

Due to the complexity of the pathophysiology behind critical illness, most commonly, a multi-parameter integration will lead to the most comprehensive evaluation of the patients’ clinical conditions.

Darmon et al. [6] proposed a model combining RRI and clinical and laboratory parameters to identify AKI reversibility in critically ill patients; this model, including RRI, urinary output, and serum creatinine, was superior to any single parameter for predicting renal recovery. Similarly, Oliveira et al. [17] proposed a model based on RRI, mean arterial pressure, and serum lactate to predict AKI in critically ill patients: this model showed excellent predictive performance, suggesting that RRI, when combined with other clinical and laboratory parameters, can provide an even better risk stratification.

## 7. Limitations and Challenges

The most important limitation of RRI measurement is its operator dependency. This need for technical skills can restrict the broader translation of RRI into clinical practice, especially in settings and areas where experienced sonographers may not be available. In addition, RRI can also be influenced by the type and quality of ultrasound equipment. Compared with older or portable devices, high-end machines with higher resolution and more sensitive Doppler capabilities may yield more accurate and reproducible findings [2]. Differences in equipment quality may therefore cause heterogeneity in RRI values reported in clinical settings or research studies.

Technical standardization of RRI measurement is another issue. Although there are general recommendations for the specific vessels [interlobar, arcuate, or segmental arteries] and number of measurements taken and how these measurements are averaged [10], there is often a certain degree of variability. This variability can complicate comparisons of results across studies or even clinical practices.

RRI might be affected by patient-specific factors such as age. For example, RRI becomes higher with advanced age, even without renal disease [64], because vascular compliance decreases as age increases. Cardiovascular factors such as heart rate, blood pressure, and cardiac output can also affect RRI. Tachycardia can lead to an underestimation of RRI while bradycardia can cause an overestimation [65]. Likewise, blood pressure variations may affect RRI, where a rise in systemic blood pressure could provoke an RRI [66] decrease. In critically ill patients, these hemodynamic effects may be crucial if we consider the fast and dramatic changes in these parameters.

Mechanical ventilation settings can substantially influence the measurement of RRI. PEEP increases RRI, presumably from the mechanical interaction between intrathoracic pressure and venous return [27]. This is especially troublesome in ARDS patients, where high PEEP levels are frequently employed, and the incremental PEEP-induced changes could mislead the meaning of the changes in RRI.

In addition, pre-existing renal conditions affect RRI. Chronic kidney disease, renal artery stenosis, and other structural renal abnormalities may all cause baseline increases in RRI that may alter the real acute changes [45,46,47,67]. This aspect is especially crucial in multi-organ dysfunction [as commonly occurs in critically ill patients] and therefore RRI. In such complex scenarios, alterations in RRI might reflect renal alterations but also systemic effects of critical illness. Changes in RRI in patients with both ARDS and AKI, such as those with sepsis, could be related to a combination of systemic inflammation, cardiac output changes, mechanical ventilation effects, and intrinsic renal disease [68]. Likewise, in patients with concomitant heart and kidney dysfunction, changes in RRI might not be so easy to understand. Rising RRI might indicate worsening heart function, progression of renal damage, or both [33,35]. Moreover, RRI is a dynamic index that can also rapidly change over time under the influence of many factors.

In summary, despite its limitations and challenges, renal RRI holds promise as a valuable tool in critical care. It offers a novel advantage over existing invasive renal hemodynamic monitoring methods by providing real-time information. However, clinicians and researchers must be mindful of these limitations and interpret RRI results within a broader context, incorporating other clinical and laboratory parameters. Overcoming these limitations will be crucial for fully realizing the potential benefits of RRI in critical care. This includes the development of standardized protocols for RRI assessment, conducting larger studies to elucidate the interactions between individual physiological factors and RRI, and exploring how RRI can be integrated with other clinical and biochemical markers to enhance its utility in complex multi-organ dysfunction scenarios.

## 8. Future Directions

One of the primary research priorities moving forward is the standardization of measurement techniques. Standardized protocols for RRI measurement need to be developed and validated, including the identification of optimal measurement sites, the determination of the appropriate number of measurements and the averaging procedure, and the standardization of equipment settings. Implementing a uniform data acquisition approach would substantially enhance the reproducibility of RRI measurements across different operators and clinical environments, thereby strengthening its utility as both a research and clinical tool [19].

Further research could come from technical innovations through the development of automated RRI measurement methods and novel ultrasound technologies to enhance the accuracy and precision of RRI measurements. Post-operative renal perfusion could be monitored continuously on a real-time basis, and continuous RRI monitoring technologies [18] might offer insight into RRI assessment in critical care settings.

The first study by Samoni et al. includes intraparenchymal renal resistive index variation (IRRIV) based on a pole-to-pole assessment of renal functional reserve (RFR) [69], an excellent indicator of the dynamic functional reserve of the kidney expressed as the range of RRI values between the maximum and minimum throughout a given time period, with the more subtle changes in RRI correlating closely with changes in renal perfusion. IRRIV was initially tested in healthy volunteers but has potential utility in patients with ARDS or HF. It could help in the early diagnosis of AKI and in decisions about renal replacement therapy and could provide more precise information about renal function in complicated hemodynamic conditions. Future studies are needed to validate this concept in critically ill patients.

Associations between RRI and other clinical and laboratory variables is also a potential topic for future investigation. AI and machine learning algorithms might add RRI to other patient data, providing a more complete evaluation. Finally, the assessment of RRI along with new kidney injury biomarkers may help to enhance risk stratification approaches [70,71].

Further studies, however, are needed to understand what influences RRI, for example, the determination of age-specific and comorbidity-specific reference ranges for RRI. Hence, we could identify some clues to detect genetic factors that may contribute to RRI and to the effect of therapies.

RRI research also needs longitudinal studies to assess the prognostic significance of time-dependent RRI changes or to assess the effect of RRI-based management strategies on long-term outcomes or the effect of acute changes in RRI on long-term renal function [4].

Interventional studies are required to address whether RRI-guided fluid management protocols are superior to standard protocols in patients with ARDS or HF. Renal replacement therapy guided by RRI and RRI-defined ventilation strategies in patients with ARDS [40] also are worthy of interest.

In multi-organ dysfunction, further studies should be conducted to determine the relative contributions of individual organs to changes in RRI. [65]. Further, while the majority of RRI research has been conducted with adults, accurately determining of age-dependent ranges for RRI in pediatric and neonatal populations will enable auspicious use of RRI in fluid management of critically ill children and enhance the applicability of RRI for assessment of renal function in premature infants [72].

Finally, the cost-effectiveness of RRI needs to be assessed in critical care settings [13].

## 9. Conclusions

Recent studies have identified RRI as a promising non-invasive tool that provides valuable insights into renal hemodynamic status in patients with ARDS and HF. RRI has demonstrated its utility in risk stratification, fluid management, and treatment response assessment, with associations noted between RRI and disease severity, oxygenation parameters, and the risk of acute kidney injury.

While RRI holds significant potential in critical care settings, several challenges hinder its broader application, including operator dependence, the need for standardized measurement protocols, and difficulties in interpretation, particularly in the context of multi-organ dysfunction. Despite these challenges, there remains considerable potential for enhancing the utility of RRI through future research aimed at addressing these limitations.

## Figures and Tables

**Table 1:** Main studies that have evaluated the prognostic significance of RRI in ARDS.

Authors	Country	Sample Size	Study Design	Aim	Outcome
Fogagnolo et al. (2022) [36]	Italia	30	Prospective, observational pilot study	To compare RRI and renal venous flow (RVF) in ARDS patients with SARS-CoV-2 and in patients with ARDS due to other etiologies.	Patients with SARS-COV-2 ARDS had higher RRI values than patients with ARDS (0.71 [0.67–0.78] vs. 0.64 [0.60–0.74], *p* = 0.04). A linear correlation was found between PEEP and RRI in patients with SARS-COV-2 ARDS (r^2^ = 0.31; *p* = 0.03) but not in patients with ARDS. A more pronounced impairment in renal blood flow was found in mechanically ventilated patients with SARS-COV-2 ARDS compared with patients with “classical” ARDS.
Giustiniano et al. (2021) [37]	Italy	105	Prospective	To analyze RRI and other hemodynamic, respiratory, and inflammation parameters in critically ill patients affected by severe COVID-19 with ARDS aiming at verifying their modifications during supine and prone positioning and any mutual correlations or interplay with RRI.	Before and during prone positioning, none of the analyzed parameters changed significantly. Pearson’s correlation coefficient showed a moderate association between CRP and RRI (r = 0.496; 95% CI, 0.165–0.726) (*p* = 0.005) and between RRI and creatinine (r = 0.425; 95% CI, 0.077–0.681) (*p* = 0.019). CRP and creatinine showed a weak correlation (r = 0.321; 95% CI, −0.059–0.620) (*p* = 0.096).
Cruz et al. (2021) [38]	Mexico	65	Prospective	To determine the role of RRI in predicting AKI and adverse outcomes in critically ill patients with COVID-19.	Of the patients who developed AKI, 68% had RRI ≥ 0.7. In addition, 75% of the patients who required RRT had RRI ≥ 0.7. In the adjusted Cox model, an RRI ≥ 0.7 was associated with higher mortality (HR 2.86, 95% CI: 1.19–6.82, *p* = 0.01).

**Table 2:** Main studies that have evaluated the prognostic significance of RRI in HF.

Authors	Country	Sample Size	Study Design	Aim	Outcome
Nijst et al.(2017) [8]	Belgium	56	Prospective	To assess the intrarenal flow in heart failure (HF) patients during the transition from euvolemia to intravascular volume overload and the relationship between intrarenal flow and diuretic efficiency.	In response to volume expansion, VII increased significantly in HFrEF patients (0.4 ± 0.3 to 0.7 ± 0.2; *p* < 0.001) and in HFpEF patients (0.4 ± 0.3 to 0.7 ± 0.2; *p* = 0.002) but not in healthy subjects (0.2 ± 0.2 to 0.3 ± 0.1; *p* = 0.622). This outcome was reversed after loop diuretic administration. In contrast, RRI did not change significantly after volume expansion.
Iacoviello et al. (2016) [32]	Italy	266	Prospective, observational	To verify the value of RRI as a predictor of worsening renal function (WRF) in a group of chronic heart failure (CHF) outpatients.	RRI was associated with WRF in a univariate (OR: 1.13; 95% CI: 1.07–1.20) as well as in a forward stepwise multivariate logistic regression analysis (OR: 1.09; 95% CI: 1.03–1.16; *p* = 0.005) including the other univariate predictors.
Geraci et al.(2019) [33]	Italy	130	Retrospective	To examine the associations between renal hemodynamics, coronary atherosclerotic burden, and carotid atherosclerotic disease.	Intrarenal vascular alterations were significantly correlated with coronary artery disease in patients with low atherosclerotic burden (GS ≤ 30 or cIMT ≤ 0.90 mm), whereas this association was not identified in those with severe coronary artery disease (GS > 30).
Ciccone et al. (2014) [35]	Italy	250	Prospective	To evaluate the clinical correlates of RRI in a group of outpatients affected by CHF as well as its role in predicting HF progression.	RRI was associated with events in a univariate [hazard ratio (HR) 1.14; 95% confidence interval (CI) 1.09–1.19; *p* < 0.001] as well as in a multivariate Cox regression analysis (HR 1.08; 95% CI 1.02–1.13; *p* = 0.004).
Ennezat et al. (2011) [48]	France	180	Prospective	To investigate the clinical relevance of intrarenal vascular hemodynamics in assessing the prognostic value of RRI for all-cause mortality or hospitalization for HF decompensation.	Mean RRI was an independent predictor of poor outcomes [hazard ratio = 1.06 95% confidence interval (1.01–1.10), *p* = 0.007] and remained significantly associated with the outcome after adjustment for univariate predictors that included low mean blood pressure, low hemoglobin concentration, and low glomerular filtration rate.
Tomii et al. (2021) [52]	Japan	330	Retrospective	To investigate the determining factors of RRI in HF patients with preserved ejection fraction (HFpEF) and with reduced EF (HFrEF).	Cox proportional hazard analysis revealed an association of RRI with the composite outcome in both HFrEF (HR 1.08; 95% CI 1.03–1.14) and HFpEF (HR 1.07; 95% CI 1.03–1.12) without an interaction (*p* for interaction = 0.770).
